# 低剂量螺旋CT在肺癌筛查中的应用

**DOI:** 10.3779/j.issn.1009-3419.2022.101.40

**Published:** 2022-09-20

**Authors:** 令芹 孔, 晓敏 张, 西川 李, 延军 苏

**Affiliations:** 1 272007 济宁，济宁肿瘤医院放射科 Department of Radiology, Jining Cancer Hospital, Jining 272007, China; 2 300387 天津，天津师范大学生命科学学院，天津市动植物抗性重点实验室 Tianjin Key Laboratory of Animal and Plant Resistance, College of Life Sciences, Tianjin Normal University, Tianjin 300387, China; 3 300060 天津，天津医科大学肿瘤医院肺部肿瘤科，天津市肿瘤防治重点实验室 Key Laboratory of Cancer Prevention and Therapy, Department of Lung Cancer, Tianjin Medical University Cancer Institute and Hospital, Tianjin 300060, China

**Keywords:** 肺肿瘤, 低剂量螺旋计算机断层扫描, 筛查, 早诊, 肺结节管理, Lung neoplasms, Low-dose spiral computed tomography, Screening, Early detection, Lung nodule management

## Abstract

肺癌是全世界发病率和死亡率最高的恶性肿瘤之一，患者的早期诊断率低、预后差，已造成严重的社会负担。通过低剂量螺旋计算机断层扫描（low-dose spiral computed tomography, LDCT）对高危人群进行定期筛查的方法能够显著提高肺癌的早期诊断率，为肺癌的诊疗带来了新机遇。近年来，全世界多个国家开展了LDCT肺癌筛查项目并取得了良好的效果，但在筛查主体的选择、筛查频率、成本效能等方面也存在一些争议。本文将从LDCT肺癌筛查的关键因素、筛查效果、肺结节的管理和人工智能对于LDCT的发展贡献等方面进行综述，探讨LDCT在肺癌筛查中的应用进展。

## Abstract (en)

Lung cancer is one of the malignant tumors with the highest morbidity and mortality in the world. The low early diagnosis rate and poor prognosis of patients have caused serious social burden. Regular screening of high-risk population by low-dose spiral computed tomography (LDCT) can significantly improve the early diagnosis rate of lung cancer and bring new opportunities for the diagnosis and treatment of lung cancer. In recent years, LDCT lung cancer screening programs have been carried out in many countries around the world and achieved good results, but there are still some controversies in the selection of screening subjects, screening frequency, cost effectiveness and other aspects. In this paper, the key factors of LDCT lung cancer screening, screening effect, pulmonary nodule management and artificial intelligence contribution to the development of LDCT will be reviewed, and the application progress of LDCT in lung cancer screening will be discussed.

肺癌是全世界发病率和死亡率最高的恶性肿瘤之一，美国癌症协会（American Cancer Society, ACS）统计数据^[1]^显示，2020年全球新发病例中肺癌占比11.4%，死亡病例中肺癌占比18%。初次就诊时已处于中晚期阶段的肺癌患者约为53.3%，错过早期最佳治疗时间，导致肺癌5年生存率仅为15.6%-16.6%^[2-4]^。因此，肺癌筛查对提高早诊率、降低死亡率以及把握最佳治疗时间等具有重要意义。

已有研究^[5]^显示，胸部X射线（chest X-ray, CXR）和痰液细胞学检查能够检出中晚期肺癌，但其对早期肺癌的检出率较低，不能有效提高患者生存率。随后有研究^[3, 6]^发现，低剂量螺旋计算机断层扫描（low-dose spiral computed tomography, LDCT）能够比CXR更高效地进行肺癌早期筛查，对于肺癌的早诊早治具有重要意义。因此，本文将从LDCT肺癌筛查的关键因素、筛查效果、肺结节的管理和人工智能（artificial intelligence, AI）对于LDCT的发展贡献等方面进行综述。

## 1 LDCT肺癌筛查简介与关键因素

### 1.1 LDCT的发展历程

1990年Naidich等^[7]^最早提出将LDCT应用于肺癌高风险人群的筛查，1992年国际早期肺癌行动计划（International Early Lung Cancer Program, I-ELCAP）^[8]^正式启动，该项目共招募了1, 000名志愿者，通过LDCT筛查发现233名受试者存在非钙化结节，随后通过活检发现27例恶性病变；而CXR仅能检测到23例受试者存在非钙化结节，其中4例为恶性病变；这项实验充分证明了LDCT的灵敏度远高于CXR。2004年美国国家肺癌筛查实验（National Lung Screening Trial, NLST）^[9]^证实，采用LDCT对于肺癌高风险人群的筛查可以有效降低肺癌的死亡率。随后荷兰-比利时肺癌筛查实验（Dutch-Belgian Lung Cancer Screening trial, NELSON）^[10]^对受试者进行了4轮筛查，并对筛查间隔进行了初步探究。随后的意大利多中心肺部检测（The Multicentric Italian Lung Detection, MILD）实验结果^[11]^表明，与NLST试验相比，持续5年以上筛查时间可以更好地降低肺癌死亡率。基于国外多项LDCT肺癌筛查的研究结果以及国内LDCT肺癌筛查的研究实践，中国近年来将LDCT应用于肺癌筛查，并制定了《中国肺癌低剂量螺旋CT筛查指南》^[12]^。

### 1.2 LDCT筛查主体的选择

目前全球开展的肺癌筛查试验中高危人群的选择方式主要有两种^[3]^：一种是基于年龄、吸烟史等高危因素进行的肺癌筛查试验，例如NLST与NELSON招募受试者的年龄处于50岁-74岁，主要为吸烟者或有过吸烟史等高风险人群^[9, 10]^；另一种是根据肺癌风险模型进行预测，例如英国肺癌筛查实验（UK Lung Cancer Screening, UKLS）是通过利物浦肺项目（Liverpool Lung Project, LLP）风险模型筛选高危人群作为受试者，该项目纳入了年龄、性别、恶性肿瘤史、吸烟持续时间、肺癌家族史（包括发病年龄）、职业暴露史以及肺炎史等危险因素来预测5年肺癌风险^[13, 14]^。由于不同的高危人群筛选模型是基于不同的预测变量实现的，最终得到的预测效果具有很大的差异；因此在肺癌筛查时要根据实际情况选择合适的筛查主体。

### 1.3 LDCT的辐射剂量风险及筛查频率

有研究^[15]^评估每2, 500名受试者中约有1例因辐射而导致癌症，因此部分专家认为LDCT的辐射可能会导致受试者具有患癌风险。为明确LDCT的辐射剂量，研究者对多项随机对照实验（randomized controlled trials, RCT）提供的CT扫描数据进行了分析；结果显示，标准胸部CT检查的平均剂量为7.0 mSv，LDCT的平均有效剂量为1.4 mSv-1.6 mSv，NLST受试者平均每人3年内累计辐射量共为8 mSv^[16]^。此外，有研究^[15]^显示NLST对于肺癌的预防效果要远大于辐射风险，且辐射风险在10年-20年之后才会显现出来。

较短的筛查间隔可能会导致辐射风险、成本以及假阳性结果的增加，而较长的筛查间隔可能会导致肺癌检出率的减少。因此，筛查间隔的选择在很大程度上会影响LDCT肺癌筛查的结果。NLST的筛查每间隔1年进行一次，共计3轮筛查^[9]^；NELSON使用的筛查间隔策略为1年、2年及2.5年，在间隔2.5年后，检测到的间歇性癌症明显多于其他两组间隔时间，且检测到的晚期癌症也明显增多，因此研究者^[10]^认为2.5年筛查间隔会导致筛查的有效性降低。年度筛查与2年筛查的使用仍然存在争议，为比较不同肺癌筛查间隔的效果，MILD试验对年度筛查与2年筛查进行了比较，数据显示两种筛查策略中间歇性癌症的数量并无明显差异^[17]^。2022年《中华医学肺癌筛查指南》建议肺癌筛查的间隔为1年，不推荐大于2年的筛查间隔，对于年度筛查正常者，建议每1年-2年继续筛查。筛查的频率影响着筛查的效能，最佳筛查间隔仍需进一步探索。

### 1.4 LDCT肺癌筛查的被接受度及筛查意识

在LDCT肺癌筛查试验中，受试者的招募和加入率是一项不可低估的挑战；社会经济地位、家族肺癌史、年龄、性别以及吸烟状况等因素都会影响参与者的依从性。Ali等^[18]^调查了UKLS试验中受试者减少的原因，调查结果显示，拒绝坚持肺癌筛查的主要是女性、65岁以上、正在吸烟、社会经济地位较低以及情感风险感知高的人群。Lopez-Olivo等^[19]^对美国报告的肺癌筛查试验中受试者依从性进行了*meta*分析，作者共纳入了15项研究，发现在受试者中，有过吸烟史的受试者人群坚持肺癌筛查的可能性大于正在吸烟者；白种人、年龄处于65岁-73岁之间人群以及接受高等教育人群坚持肺癌筛查的可能性更大。

Raz等^[20]^对1, 384名医护工作人员对肺癌筛查指南的了解程度进行了随机抽样调查，47%的医护工作者对肺癌筛查指南认知较高，且在认知程度较高的医护工作人群中，97%认可LDCT肺癌筛查可有效降低肺癌死亡率这一结果。Iaccarino等^[21]^对肺病学专家提供肺癌筛查的倾向以及对LDCT肺癌筛查的态度进行了调查评估，数据显示52.4%的肺病学专家对肺癌筛查指南表示认可，22.7%的专家认为LDCT肺癌筛查存在过度诊断，24.9%专家认为筛查不足。因此提高医护人员对于肺癌筛查指南的了解程度有助于LDCT肺癌筛查的推广。

### 1.5 LDCT肺癌筛查的成本效能

应用LDCT进行肺癌筛查时不仅要考虑安全性和准确性，也要考虑其经济学效益。Snowsill等^[22]^对LDCT应用于肺癌高危人群筛查进行了临床有效性以及经济效益的评估，发现在常规阈值下，单次筛查是具有成本效益的，但对成本的影响和收益的大小仍存在很大的不确定性。2020年，Du等^[23]^评估了在高危人群中使用LDCT进行肺癌筛查的成本效益，结果显示使用LDCT在55岁-80岁的重度吸烟男性中每年进行一次肺癌筛查与在50岁-80岁的重度吸烟女性中每两年进行一次肺癌筛查，成本效益最高。Criss等^[24]^根据美国预防服务工作组（U.S. Preventive Services Task Force, USPSTF）（80岁）、医疗保险和医疗补助服务中心（Centers for Medicare & Medicaid Services, CMS）（77岁）和NLST（74岁）的停止肺癌筛查最大年龄建议，开发了4个独立微观模拟模型评估了LDCT的成本效益，虽然存在潜在的不确定性，但NLST和CMS筛查策略具有很高的成本效益。此外，Wade等^[25]^以NLST选择人群的标准作为基础，评估了LDCT肺癌筛查的成本效益，结果发现LDCT肺癌筛查具有成本效益的可能性较低。

以上数据证明，LDCT肺癌筛查的成本效益因实施筛查的国家地区而异，应对不同的筛查结果、筛查对象采取精准的筛查措施，仍需要进一步探索其可行性。

## 2 LDCT在肺癌筛查中的效果

### 2.1 提高肺癌的早期诊断率

在NLST中，LDCT检出的肺癌中Ⅰ期肺癌占54%；CXR检出的肺癌中Ⅰ期肺癌占37.8%^[26]^。在Yang等^[27]^的基线筛查中，LDCT检出的肺癌患者中早期肺癌占94.1%，在常规护理组检出的肺癌中早期肺癌占20%。有研究者对4, 690例无症状受试者通过LDCT进行了肺癌筛查，结果显示，除局限期小细胞肺癌以外，Ⅰ期肺癌的检出率为76%^[28]^。以上数据说明，LDCT筛查能够提高早期肺癌的诊断率，而早期诊断率的提高有利于患者及时接受治疗。

### 2.2 降低肺癌的死亡率

目前全球各地开展的多项肺癌筛查试验证明，LDCT筛查能够有效降低肺癌的死亡率。NLST是目前规模最大的LDCT肺癌筛查试验，该试验报告首次提出，与CXR筛查组相比，LDCT筛查组的死亡率下降了6.7%^[9]^。NELSON在2020年公布的最终结果^[29]^中显示，与未接受筛查人群相比，LDCT筛查组10年累计肺癌死亡率仅为0.76%，明显低于未接受肺癌筛查患者。

MILD试验^[11]^对LDCT筛查组和对照组进行了比较，数据显示，与对照组相比，LDCT筛查组在第5年时肺癌死亡风险降低了58%，总体死亡率降低了32%；在第10年死亡风险降低了39%，总体死亡率降低了20%，与NLST试验相比，MILD试验证实了延长筛查时间超过5年可进一步实现总体死亡率和肺癌死亡风险的降低。在德国肺癌筛查干预（German Lung cancer Screening Intervention, LUSI）试验中通过性别建模比对发现，经LDCT筛查的女性患者的肺癌死亡率降低，得出了LDCT筛查降低死亡率的结论^[3, 30]^。但也有一些RCT试验^[13, 15]^显示，与无干预组、CXR筛查组相比，LCDT筛查组的肺癌死亡率降低并不显著。

### 2.3 提高戒烟意识

肺癌筛查不能取代戒烟等预防措施，但仅靠预防措施无法达到降低肺癌死亡率的目的。欧盟立场声明（European Union Position Statement, EUPS）曾对NLST提出建议，希望研究者对参与肺癌筛查的人群提出戒烟指导^[31]^。Tanner等^[32]^对NLST的筛查数据进行了二次分析，实验数据显示，与具有吸烟史的受试者相比，吸烟者的肺癌死亡风险与总死亡率增加；戒烟7年的受试者死亡率降低了20%；戒烟15年并坚持LDCT筛查的受试者，肺癌死亡风险降低了38%。Pistelli等^[33]^在随机ITALUNG肺癌筛查试验中发现，参与LDCT肺癌筛查的受试者戒烟率明显高于对照组，肺结节的存在以及戒烟计划的实施能够提高戒烟率。在UKLS试验中的研究^[34]^发现，与对照组相比，额外的检查能够提高受试者的戒烟率。Ashraf等^[35]^对丹麦肺癌筛查试验（Danish Lung Cancer Screening Trial, DLCST）中受试者的吸烟习惯进行了调查分析，发现在经过5轮LDCT肺癌筛查后，戒烟率增加了13%且戒烟比较彻底。

## 3 肺结节的管理

肺结节的管理影响着肺癌筛查的效果。已有的LDCT肺癌筛查试验的数据^[36]^显示，约50%的受试者在肺癌筛查中检测到结节的数量≥1个。在NELSON试验中，接受LDCT筛查的受试者中有5%-7%在后续随访中被检测到有新的结节^[10]^。研究^[37, 38]^证明，罹患肺癌的概率与新生结节的数量和大小无关，被检测到的单个结节即使体积较小（体积 < 50 mm^3^或最大直径 < 5 mm），也有同样高风险转化为恶性肿瘤。目前全球对于阳性结节的定义以及肺结节的管理没有明确的定义，主要根据大小、生长速度和类型对其进行评估。

2018年我国肺癌筛查指南将实性结节或部分实性结节直径≥5 mm、非实性结节≥8 mm定义为阳性结节，根据阳性结节的大小确定随访原则及判断其是否需要进行临床干预^[39]^。NLST在基线筛查时将最大直径≥4 mm的肺结节定义为阳性，然而NLST报告了大量的假阳性结果；NELSON报告^[38]^称研究者通过测量结节体积减少了假阳性结果的产生，此后，通过体积大小定义阳性结节被应用于EUPS、NELSON-Plus等筛查试验中。

实性结节主要包括钙化结节和非钙化结节两类，非钙化结节又分为固体和亚固体。实性结节在肺癌筛查中比较普遍，亚固体结节通常发现于肺癌前期或早期肺癌中，但其与较高的恶性肿瘤风险相关^[38, 40]^。此外，结节转化为恶性肿瘤风险还可能与其附着位置有关，NELSON试验^[41]^中，82.2%的腺癌在肺的外围被检测到，而在肺的中央仅检测到17.8%，此外，45%的肺癌在肺右上叶被检测到。

肺结节管理过程中，假阳性与惰性肿瘤的出现均会影响肺癌筛查的结果。因此，为确保LDCT肺癌筛查的结果更加准确有效，应建立更加全面的评估标准用于肺结节的管理。

## 4 AI对于LDCT的发展贡献

大范围内实施肺癌筛查无疑会给放射科医生带来巨大工作增量。近年来，AI系统逐渐作为第一读者、第二阅读器或并发阅读器等应用于肺癌筛查，并取得了较好的效果。在减少放射科医生工作量方面，AI作为第一读者是最佳策略，放射科医生可以只审查AI系统具有临床意义的结节，但此方法存在着较大的假阴性风险^[13]^；计算机辅助检测系统（computer-aided detection, CAD）作为放射科医生的第二阅读器可以提高结节检测的准确性，与传统阅读器相比，CAD用作肺结节的检测可以将平均敏感度由63%大幅提高至76%^[42, 43]^。Silva等^[43]^研究了在MILD实验中使用CAD检测亚固体结节的情况，研究表明CAD的灵敏度高于人类视觉阅读，但仍需要人工对CAD标记的结节进行人工视觉确认，以减少假阴性或假阳性结果的产生，该研究同样证明了CAD与人类视觉阅读结合可以大幅度提高肺结节检测的效率及准确性。

## 5 小结与展望

肺癌作为发病率和致死率极高的恶性肿瘤之一，严重危害我国人民的生命健康。LDCT肺癌筛查为我国肺癌的早诊早治提供了新机遇，并取得了良好的效果，对于提高国民健康具有重要意义。但中国人口众多，经济差异、地域差异以及人文差异为肺癌筛查的普及带来了较大的阻力。此外，LDCT肺癌筛查仍存在过度诊断、假阳性、筛查间隔以及成本效益等问题亟待解决，其用于大规模筛查仍然需要不断探索更加合理科学的筛查制度。随着各项生物科学技术、检测技术的日趋成熟以及AI的不断发展，LDCT肺癌筛查的准确率将不断提高，将为提高肺癌患者早诊率、降低死亡率、减轻国民就医压力和社会负担提供巨大帮助。
